# Interface Design for CMOS-Integrated Electrochemical Impedance Spectroscopy (EIS) Biosensors

**DOI:** 10.3390/s121114467

**Published:** 2012-10-29

**Authors:** Arun Manickam, Christopher Andrew Johnson, Sam Kavusi, Arjang Hassibi

**Affiliations:** 1 Department of Electrical and Computer Engineering, University of Texas at Austin, Austin, TX 78712, USA; E-Mail: arjang@mail.utexas.edu; 2 Robert Bosch Research and Technology Center, Palo Alto, CA 94306, USA; E-Mails: christopher.johnson2@us.bosch.com (C.A.J.); sam.kavusi@us.bosch.com (S.K.)

**Keywords:** Electrochemical Impedance Spectroscopy, integrated biosensors, CMOS integrated circuit, label-free, real-time, microarrays

## Abstract

Electrochemical Impedance Spectroscopy (EIS) is a powerful electrochemical technique to detect biomolecules. EIS has the potential of carrying out label-free and real-time detection, and in addition, can be easily implemented using electronic integrated circuits (ICs) that are built through standard semiconductor fabrication processes. This paper focuses on the various design and optimization aspects of EIS ICs, particularly the bio-to-semiconductor interface design. We discuss, in detail, considerations such as the choice of the electrode surface in view of IC manufacturing, surface linkers, and development of optimal bio-molecular detection protocols. We also report experimental results, using both macro- and micro-electrodes to demonstrate the design trade-offs and ultimately validate our optimization procedures.

## 1. Introduction

Biomolecular detection sensors (*i.e.*, biosensors) are used for detecting a wide variety of molecules such as DNA, proteins, toxins and even micro-organisms [1] and [2]. Due to their detection versatility, biosensors are implemented in a variety of applications such as *in-vitro* molecular analysis, point-of-care (PoC) diagnostics, cancer research, food processing and water quality monitoring [3] and [4] . Today, the majority of these biomolecular tests are performed in core laboratory environments using bulky and complex equipments. Such instruments are generally immobile and the tests are expensive and time-consuming. Recently, there has been a push towards creating compact and integrated biomolecular detection systems, which can transfer the bio-analysis procedure from core facilities to portable PoC. In addition to the obvious small form-factor requirements for these systems, they also have to enable detection parallelism and multiplexing. The main reason for the latter is in high-performance biosensing one needs to either screen multiple analytes concurrently, or leverage parallelism redundancy to increase specificity and accuracy.

Among the various biomolecular detection methods, electrochemical impedance spectroscopy (EIS) is a powerful method, which is also compatible with integration, and, in theory, can be used for the detection of a wide variety of biomolecules [5] and [6]. One advantage of EIS over other methods is that it offers allows label-free and real-time detection by relying on the measurement of small changes in the electrode-electrolyte interface impedance in order to detect and quantify probe-analyte interactions. EIS does not require any molecular modifications or labels, as is required in other systems such as the fluorescent-based or magnetic-based biosensors [7–9]. Furthermore, it is possible to monitor the impedance changes that occur in real-time, *i.e.*, as the surface binding occurs. This is in contrast to most other biosensors that can only offer end-point measurements and require the binding interactions to be stopped (and the sample to be removed) before quantifying the probe-analyte interactions.

In spite of these advantages, there are many challenges in building an EIS-based biosensor. The sensitivity of label-free systems, so far, has been inferior compared with biosensors that use labels. In addition, the required instrumentation for EIS is complex and, today, bulky and expensive bench-top systems are an integral part of EIS platforms. These instruments typically only have a single or a few measurement channels. Hence, it is not practical to develop highly multiplexed assays using EIS, where hundreds to thousands of different analytes need to be detected in parallel. This make EIS, in its current state, not very suitable for developing compact and portable PoC biosensors .

Fully-integrated biosensor systems using semiconductor fabrication processes have been built in the past that promise unprecedented compactness, sensitivity, detection dynamic range (DDR) and cost-efficiency [7]. Reference [10] demonstrates one such work, where a fully integrated EIS IC based system was developed. However, the use of ICs brings a new set of constraints to biosensors in general and EIS-based systems in particular, such as the limited availability of surface options, semiconductor-compatible bio-functionalization, hybrid fluidic-electronic packaging and handling. Although such systems are a good candidate for next generation biosensors, they are not easy to build. The main reason is that the use of ICs brings a new set of constraints to biosensor design, and EIS-based systems are no exception. For example, limited available materials and surface options in fabrication, semiconductor-compatible bio-functionalization, hybrid fluidic-electronic packaging and handling are among some of the limiting challenges.

In this paper, we tackle the above challenges by examining various design and optimization aspects of EIS built using complementary metal-oxide-semiconductor (CMOS) processes, particularly its bio-to-semiconductor interface design. We discuss, in detail, considerations such as the choice of the electrode surface, surface linkers, and development of optimal bio-molecular detection protocols, all in view of IC manufacturing. In Section 1, we will briefly introduce the basic concepts in EIS based biosensing, by examining the equivalent circuit model and explaining how molecular binding changes the electrode-electrolyte interface impedance. In Section 2, we will present an example integrated EIS biosensor platform, and discuss the performance metrics achievable using a CMOS IC. We will be able to see that the performance metrics are superior to that of bench-top instruments. In Section 3, which focuses on the protocol development perspective of EIS-based systems, we will discuss about various surface options that are semiconductor compatible and discuss why we choose gold as an ideal choice for the electrode surface. The use of gold allows us to use thiol-based linker molecules, which form self-assembled monolayers on the gold surface. By making use of experimental data, we will discuss the impact of the length of the linker molecule on the sensitivity and surface coverage. We will present an example antigen-antibody system using macroscopic gold electrodes and discuss the experimental results obtained showing the changes in the impedance with binding. Section 4 focuses on development of the methods to functionalize the surfaces of EIS-based. Finally, in Section 5, we discuss the results obtained from the chemical and bio-molecular detection experiments.

## 2. EIS-Based Biosensor Principles

**Figure 1.**
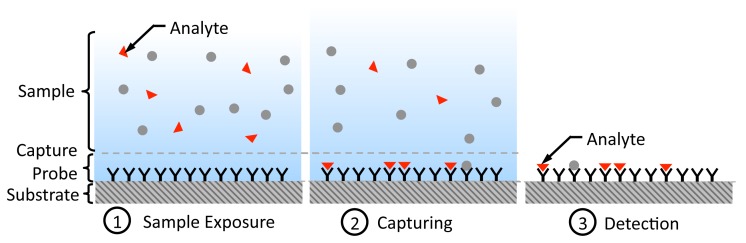
Steps in affinity-based biosensing.

Affinity-based biosensing is a fundamental method to estimate the concentration of an analyte (molecule of interest being analyzed) in an aqueous sample ([1,2,12]). They make use of biological recognition elements (also known as probes) such as DNA oligonucleotides and antibodies, which can selectively bind to the analyte. In Figure 1, we show the steps involved in an affinity-based biosensing process [12]. Initially, the capturing probe molecules are immobilized on a solid surface. Subsequently, the probes are exposed to a sample containing the analytes that can freely diffuse and hit the probes and attach to them. Finally, for detection, the number of captured analytes attached to the probes is counted in order to arrive at an estimate of the original analyte concentration.

The detection step (sometimes referred to the transduction step) is critical and is different among platforms. In this step, the signal is converted from the “molecular domain” to a measurable signal such as an optical or electrical signal. There are different methods available for detection (e.g., fluorescent [8] or magnetic-bead based [9]) where each require a different transducer. In this paper, the focus is on EIS.

**Figure 2.**
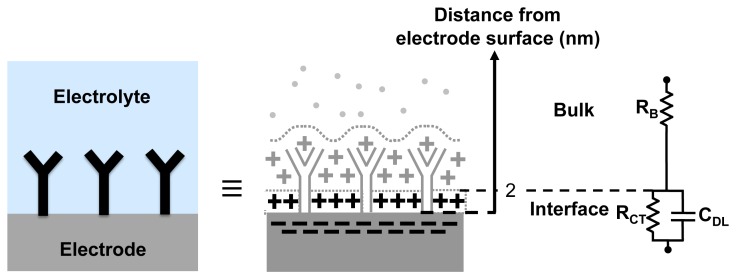
Circuit model of an electrode-electrolyte system.

In order to understand how the EIS-based biosensing works, we take advantage of an equivalent circuit model for the electrode-electrolyte interface, to relate various circuit elements to the different electrochemical phenomena. Figure 2 shows a widely-used equivalent circuit used to model the electrode-electrolyte interfaces ([5,6,13]). It consists of the bulk resistance of the solution *R_B_* in series with the impedance of the electrode-electrolyte interface. The interface impedance consists of the charge transfer resistance (*R_CT_*) in parallel with the capacitance of the double layer (*C_DL_*). The charge transfer resistance is attributed to the electrostatic interactions of ions in the solution with the electrons in the electrodes, which produced a current. A net charge is relocated from the electrode to the electrolyte or vice-versa [13]. The double layer capacitance is attributed to the spatial distribution of ions formed in the proximity of the electrode-electrolyte interface. For typical physiological buffers, the double layer extends only a few nm from the surface.

The expression for the overall admittance (*Y*) (magnitude and phase) of the electrode-electrolyte system is given by

(1) $$\left|Y(j\omega )\right|=\frac{1}{R_{CT}+R_{B}}\frac{\sqrt{1+{\left(\omega C_{DL}R_{CT}\right)}^{2}}}{\sqrt{1+{\left(\omega C_{DL}\frac{R_{CT}R_{B}}{R_{CT}+R_{B}}\right)}^{2}}}$$

(2) $$\theta (j\omega )=-\tan^{-1}(\omega C_{DL}\frac{R_{B}R_{CT}}{R_{B}+R_{CT}})+\tan^{-1}(\omega C_{DL}R_{CT}).$$

Impedance based biosensors take advantage of the changes in the interface impedance with the attachment of the analytes to the probes. The attachment of a large target onto an immobilized probe reduces the capacitance *C_DL_*, due to the increase in the thickness of the double layer and also due to reduction in the dielectric constant near the interface (e.g., *ε_r_* of water is ≈81, whereas *ε_r_* of organic molecules is around 2–3). Also, the larger molecular complexes formed can block the flow of the ions (current) through the interface, leading to an increase in *R_CT_* ([5,14,15]). Finally, the impedance can also change due to changes in the charge density in the proximity of the electrode surface, which can be caused by the ionization of molecules (for both probe and analyte).

There are multiple examples in the literature that demonstrate the utility of EIS in bio-molecular sensing [5]. For example, in the immunoassay system describe in [6], *R_CT_* changes significantly with the concentration of captured antigen. In [16] on the other hand, changes in capacitance with antigen-antibody bindings are used for sensing. It is important to realize that independent of which electrical parameter is used in probed in EIS, the binding should always happen within the interface layer (*i.e.*, the double layer). If this condition is not satisfied and the probe-analyte interactions happen outside this region, little impedance changes can be observed. This is predominately due to the high ionic concentration of biological buffers used in biosensors, which generally dominate the impedance of the bulk solution outside of the interface layer.

## 3. An Integrated CMOS EIS Biosensor

### 3.1. Biosensor Integration

**Figure 3.**
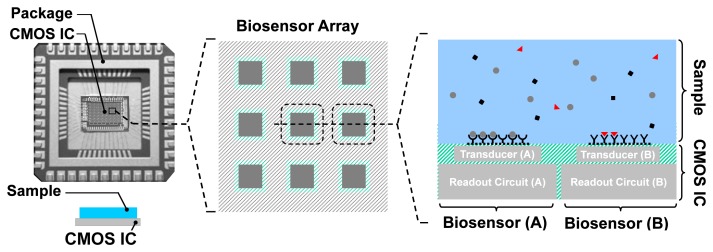
Anatomy of an integrated biosensor.

A typical setup for an integrated biosensor is shown in Figure 3. The electronic IC, fabricated in a CMOS process, is placed in a special package and the sample containing the analytes is directly placed on top of the CMOS chip. The chip, almost always, has an array of pixels, each consisting of a transduction element, along with the integrated readout circuit. The transducer is an electrode for electrochemical biosensors such as EIS-based biosensors. Depending on the application, different capturing probe molecules can be immobilized on different transduction sites, accommodating parallelism in detection. The readout circuit enables the measurements through low-noise signal amplification, signal conditioning, and to some extent signal processing. Depending on the sensor size, available silicon area, and the CMOS fabrication process used, analog-to-digital converters (ADCs) and digital signal processing (DSP) blocks can also be integrated on the same CMOS die.

### 3.2. Electrode Design

**Figure 4.**
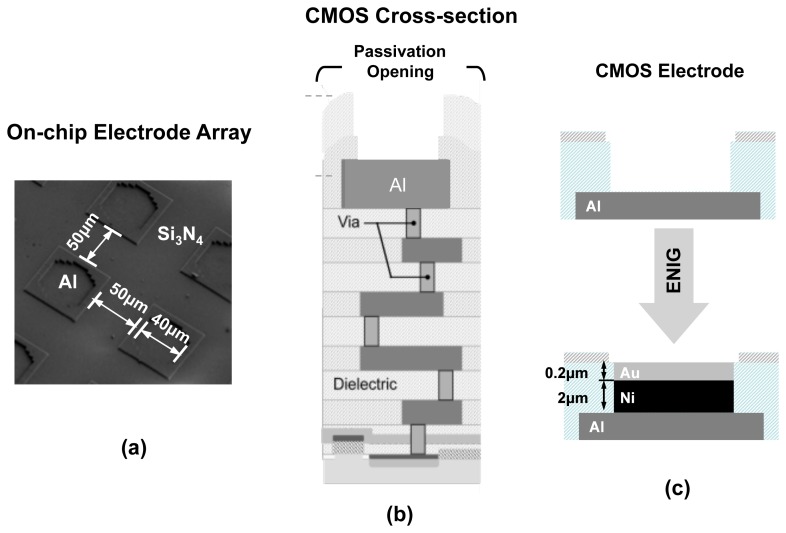
**(a)** SEM cross section of the on-chip electrode array (**b**) Cross section of CMOS process (**c**) CMOS electrode and post-processing modification with ENIG.

Since EIS requires an electrode surface to act as its transducer, we should consider enhancing the surface of electronic ICs to accommodate this requirement. The cross section of a typical CMOS chip is shown in Figure 4(b) where the transistors and active devices are created in silicon substrate and the interconnect layers (embedded in dielectric layers) on placed on top. Aluminum is the typical metal used for building the interconnects [17]. As illustrated, the easiest option to create an electrode is to use the aluminum surface of the top metal interconnect layer as the sensing electrode surface, as no additional fabrication processes steps are required. However, the usage of aluminum is generally not preferred. Aluminum surface rarely remains in its unionized state when exposed to biological buffers. Aluminum forms a protective oxide layer on its surface near neutral pH value. However, this protective coating is removed under high pH conditions and aluminum surface is heavily ionized under basic as well as acidic conditions.

Another choice is to use metal oxides such as alumina and tantalum oxide for sensing. Such metal oxides are popular in ion-sensitive field-effect transistor (ISFET) sensors. Reference [22] provides a good review of ISFET based sensors and its various applications in chemical and biomolecular sensing. However, the oxide layers prevent any DC current flowing through the interface, which can be limiting for Faradaic sensing electrodes, in which the charge transfer resistance plays a key role and the DC current flowing through the system needs to be determined.

Gold (Au) is a more suitable choice as it is stable and stays in its solid neutral state, over a wide range of potential as well as pH levels. Also, there are a number of surface immobilization protocols available for attachment of biomolecules onto a gold surface ([23,25]). These protocols take advantage of the strong thiol bond, *i.e.*, gold-sulphur bond. Thiolated molecules (molecules that have sulphur in their structure) are used in surface immobilization steps. Review paper [26] discusses how thiolated carbon chains form a well-oriented, uniform and stable blocking layer on the gold surface. These SAM layers can also be made functional [16], making them suitable for the immobilization of a wide variety of molecules, such as DNA and antibodies, onto the gold surface.

The electrodes in our design are created by first forming passivation openings on the top metal layer of the CMOS process (*i.e.*, Aluminum), as it is done for input/output (I/O) pads (illustrated in Figure 4). The exposed electrodes are approximately 40 *μm* × 40 *μm* in size and are separated by a distance of 60 *μm*. To create an universal Au electrode surface, we took advantage of an electroless nickel (Ni) immersion Au (ENIG) plating process [24] to first form a Ni layer and then a Au layer on the exposed Al electrode surfaces (see Figure 4). The rationale behind using the Ni layer first is that Au cannot be directly electro-plated on Al surfaces and an intermediate layer is generally required. The advantage of this method is that it does not require the use of lithography masks since Ni and Au do not attach to the dielectric layers. The thicknesses of the Ni and Au layers are approximately 2 *μm* and 200 *nm*, respectively After the formation of the Au surface, we take advantage of the thiol-based protocols methods to bio-functionalize it.

### 3.3. Impedance Detection

**Figure 5.**
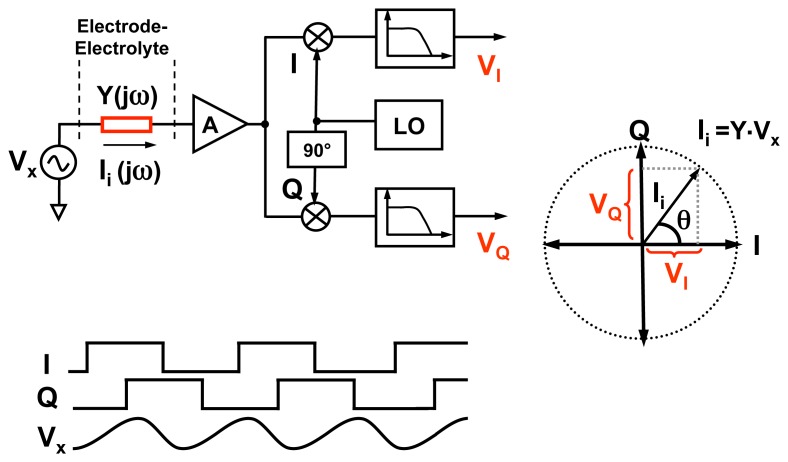
Coherent detection architecture for impedance measurement.

Different integrated electrochemical biosensors have been reported before [18–21]. However, in this paper, we use a specific EIS IC platform, which we had been previously developed. This design has on-chip electrodes, very compact pixel size and the ability to provide a large measurement dynamic range over a wide range of frequencies. The circuit implementation details of this work are presented in [10] and [11]. The goal of our detection platform is to measure, in real-time, the admittance of the electrode-electrolyte interfaces. Such a measurement is performed using lock-in detection technique (very similar to the coherent receiver technique using IC based radio frequency receivers) (shown in Figure 5). We place a large reference electrode (which is shared by all the pixels in the array) in the solution to establish the sinusoidal excitation voltage signal, *V_X_*(*ω*), across all electrodes. Next, we measure individually the current flowing through each electrode, *I*(*ω*) and compute the electrode-electrolyte admittance of the *i^th^* pixel, *Y_i_*(*ω*), using the formula

(3) $$Y_{i}(\omega )=\frac{I_{i}(\omega )}{V_{x}(\omega )}$$

where *I_i_*(*ω*) is the measured current at the *i^th^* pixel. Since all signals in this system are sinusoidal, to find *Y_i_*(*ω*), we only need to calculate the relative amplitude and phase shift of *I_i_*(*ω*) compared with *V_x_*(*ω*). The electronic detection circuit first amplifies the current *I_i_*(*ω*) in each pixel by means of a low-noise transimpedance amplifier (TIA). The TIA is an electronic circuit, which has a current-to-voltage conversion gain. The output of the TIA is then multiplied, using mixers, with orthogonal sinusoidal signals (I and Q) at the frequency *ω* (the same frequency as the excitation source). Subsequently, the higher-order harmonics (components at 2*ω*, 3*ω etc.*) are removed using a low-pass filter. The DC amplitude at output of the I and Q channels of the *i^th^* pixel, denoted by *V_I_*(*i*) and *V_Q_*(*i*) respectively, are then used to estimate the amplitude and phase of *Y_i_*(*ω*) using the formula

(4) $$\left|Y_{i}(\omega )\right|=\frac{\sqrt{V_{I}^{2}(i)+V_{Q}^{2}(i)}}{A\left|V_{x}(\omega )\right|},$$

and

(5) $$∠Y_{i}(\omega )=\tan^{-1\;}\left(\frac{V_{Q}(i)}{V_{I}(i)}\right).$$

**Figure 6.**
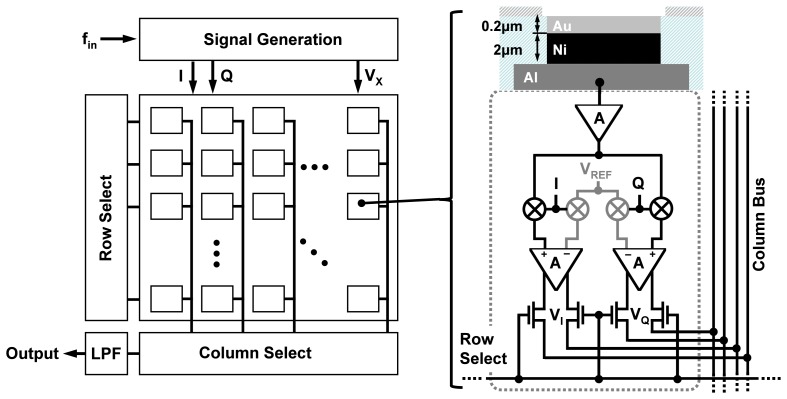
System architecture.

**Figure 7.**
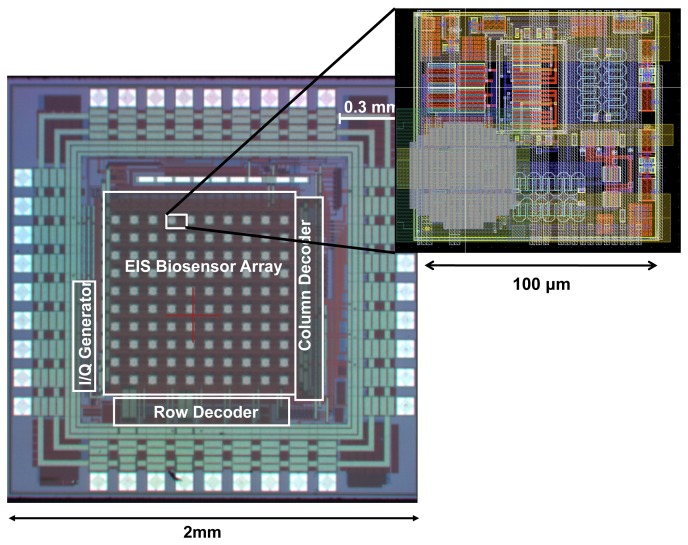
Die photograph.

The overall chip architecture is shown in Figure 6. The chip consists of an array of 10 × 10 pixels where column and row decoders are used to scan the array and access the individual outputs of pixels sequentially. This approach is similar to the readout structure of CMOS image sensors [27]. Each pixel within the array consists of a sensing electrode and the coherent detector readout circuitry, which include the low-noise TIA for current amplification and two mixers for multiplication. It is important to realize that the capturing probe of each pixel can be different and hence this particular system, with 100 pixels, can be used to detect up to 100 different analytes. The pixel circuitry occupies an area of 100 *μm* × 100 *μm* and is capable of measuring impedance over 5 orders of magnitude. Furthermore, the pixels can work in a wide range of frequencies from as low as 10 Hz to as high as 10 MHz. The pixels are capable of detecting admittance changes as small as 10 nS (which sets the sensitivity limit) and at the same time can measure up to 1 mS (which sets the upper detection limit), providing a large measurement dynamic range of 100 dB, which is superior to bench-top instruments. The chip has a 10 × 10 array of pixels, integrated in an area of 2 mm × 2 mm as shown in Figure 7.

The high frequency operation of this EIS IC platform is useful for the following reasons. As electrodes become smaller and smaller, it is possible to study the electrode-electrolyte system at higher frequencies. For a 4 *mm* × 4 *mm* electrode size, the effect of surface impedance can only been seen up to 300–1,000 Hz. In the case of 40 *μm* × 40 *μm* electrode size, the effect of surface impedance can be seen up to much higher frequencies, up to 400–500 kHz. In order to get an accurate estimate of the impedance, it is also useful to measure the bulk resistance *R_B_*, which requires the operation at frequencies greater than 1 MHz at smaller electrode sizes. Such a measurement will help us to better fit the data onto the equivalent circuit model as shown in Figure 2. Furthermore, operating the interface circuitry (front-end before the output of the mixer) at higher frequencies is advantageous in IC based systems, where the presence of low frequency flicker noise can affect the circuit performance at lower excitation frequencies.

The overall chip performance and its key metrics are listed in Table 1.

One other important performance metric is the scan rate of the array. As explained in Figure 5 and in Equations (4) and (5), the output *V_I_* and *V_Q_* are low-pass filtered in order to extract the low frequency components. The settling time of the filter sets the pixel measurement time and hence the scan rate. Lower the filter bandwidth, better is the noise performance of the system, however the scan rate is lower. For a 10 Hz bandwidth (dynamic range = 97 dB), the pixel measurement time (for a single excitation frequency) is 1.1 s. If a wider filter bandwidth such as 1 kHz is used, then the pixel measurement time is reduced, however this comes at the cost of reduced dynamic range (around 92 dB). Since the pixel outputs are connected to the chip's output one at a time, the measurement time required for a complete scan of 100 pixels (at a single excitation frequency) is 110 s (at dynamic range of 97 dB) and 1.1 s (at a dynamic range of 92 dB). For equilibrium measurements, the array scan rates in the order of 110 s is acceptable, as typically the processes such as DNA hybridization or protein attachment could take half an hour to an hour to complete. For real-time measurements, where speed is more important than precision, we can operate at much higher scan rates (1.1 s or even lower). For single pixel measurements, the scan rates can be as high as 10k samples/s.

## 4. EIS Considerations and Experiments

Before performing experiments using the CMOS chip, it is essential to understand the various considerations in building EIS experiments and to optimize bio-molecular detection protocols. Since the initial expenditure of building IC protoypes is high, we decided to perform some experiments using macroscopic gold slides. The results that we obtained from these experiments were then used optimize the experiments using the CMOS chip. In this section, we first begin with a discussion of different techniques for bio-molecular attachment onto the gold surface and later present an example label-free antigen-antibody system (immunoassay).

### 4.1. Linkage of Bio-Molecules onto Au Surface

**Figure 8.**
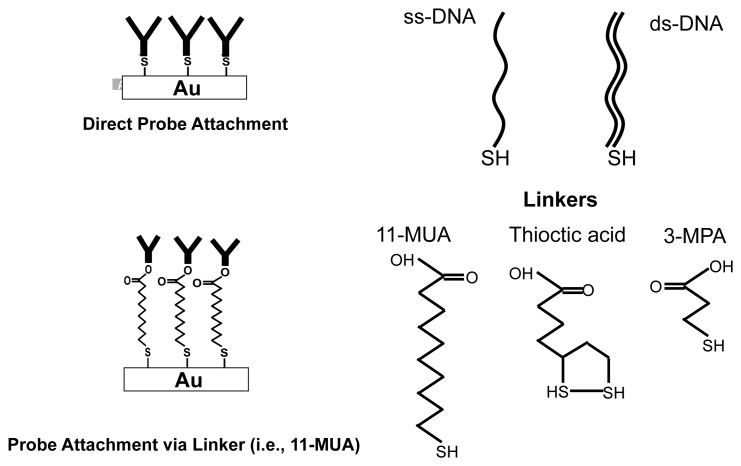
Direct and indirect attachment of molecules onto gold surfaces.

Sulphur compounds (e.g., thiol compounds) have a strong affinity to gold surfaces [26]. The binding of the SH group to the gold surface is very strong (the bond strength is approximately 10 *kT*) [26].There are two different ways of attaching molecules onto a gold surface. One is the direct attachment method, where the biomolecules are chemically modified, such that they have SH groups as their terminal. One such example is the thiolated DNA strand [28]. The terminal base (3′-end or 5′-end) of the DNA molecule is modified by a SH group as shown in Figure 8. Protein molecules can directly attach to the gold surface due to the hydrophobic and electrostatic interactions between the protein molecule and the gold surface [29]. While the direct attachment method serves as the simplest immobilization scheme, it has several disadvantages for larger molecules such as antibodies. To name a few, we can mention low surface coverage, insufficient blocking capability, denaturing probability, and surface desorption.

One way to overcome these above challenges is to take advantage of proper linker molecules. Functional alkanethiols are suitable examples. Figure 8 shows some examples of functional alkanethiols (11-MUA, 3-MPA and thioctic acid), which can serve as linkers. All of these have SH groups, which can bind to the gold surface, and a functional COOH group. This COOH group can be modified to form NHS esters, using the EDC/NHS protocol [25]. The covalent binding between the NHS ester group and the amine group in proteins is very strong (and irreversible) and hence the linker molecules ensure the stability of protein attachment to the gold surface. Furthermore, the alkanethiols form self-assembled monolayers (SAM) on the gold surface. These SAM layers have a well-defined composition, structure, and thickness. These monolayers form a pinhole free isolating molecular films. Formation of a thin, pinhole free monolayer close to the surface makes it possible to develop capacitance-based biosensors, which measure changes in the surface capacitance to perform detection [16].

### 4.2. Properties of Monolayers

One of the most important parameters in the selection of the right linker is its length. The length of the monolayer, formed from the linker molecule, has impact on sensitivity, stability and packing density [30]. In order to analyze the impact of linker length on the performance of an EIS based biosensor, we performed experiments to characterize different formed thiol based monolayers. In all experiments, the gold surface was first thoroughly cleaned (using RCA clean) and subsequently immersed in solutions containing 11-MUA, 3-MPA or thioctic acid (shown in Figure 8). The detailed experimental procedure is provided as a part of the supplementary material. EIS experiments are then performed on these surfaces, with an active area of 4 *mm* × 4 *mm*, in 1 × PBS (phosphate buffered saline) solution.

**Figure 9.**
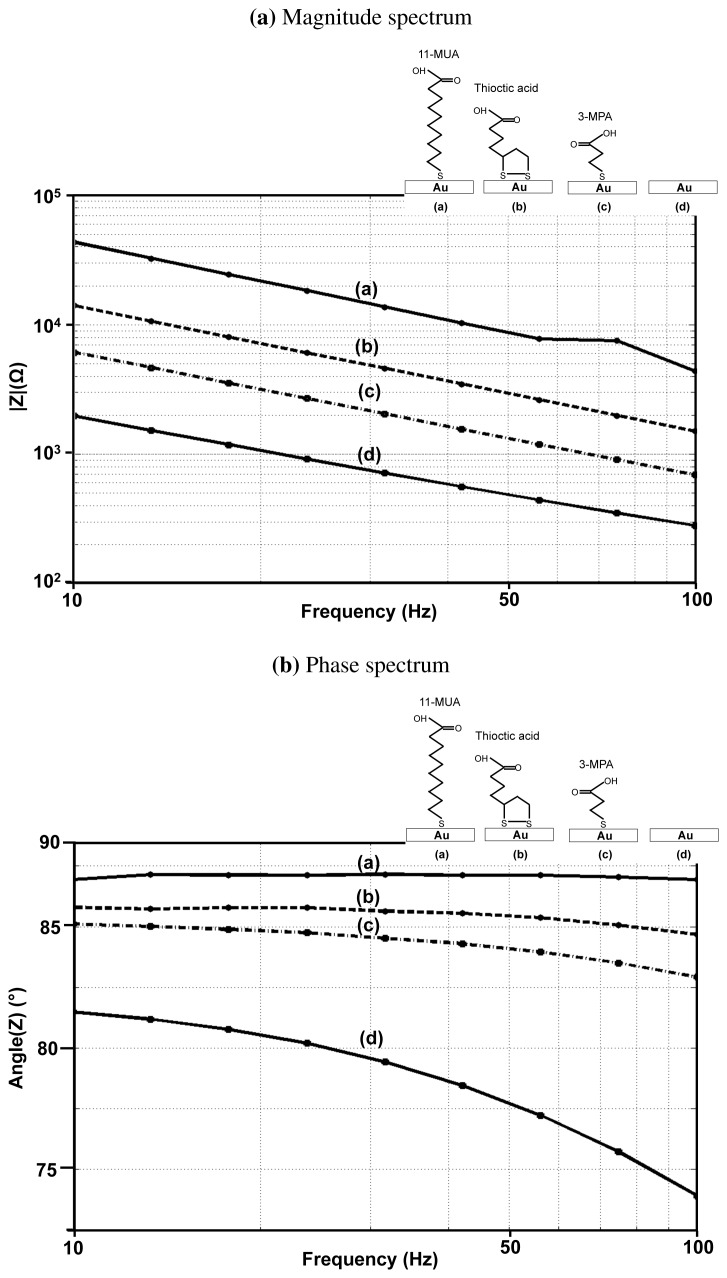
Experimental determination of the magnitude and phase spectrum of electrode-electrolyte system with different monolayers immobilized onto the gold surface (a) 11-MUA (b) thioctic acid (c) 3-MPA (d) gold. The measured c*C_DL_* values are (a) 2.48 (b) 6.6 (c) 16.5 (d) 49.73 $\frac{\mu F}{cm^{2}}$.

Figure 9(a,b) show the impedance spectra of three different monolayers (11-MUA, 3-MPA, and thioctic acid) on gold and the measured impedance spectra. The phase angle of 11-MUA monolayer is very close to 90° and, whereas for a bare gold surface, it is 78° and for 3-MPA, it is 85°. The magnitude of the capacitance is in the following order, Bare gold > 3-MPA > Thioctic acid > 11-MUA. These measurements confirm to the experimental results shown in previous studies on the impact of the thickness of the monolayer on surface coverage, surface impedance and the blocking capability of the monolayer ([31–33]). A thicker monolayer has a larger surface coverage, which is indicated by the phase angle being closer to 90°. It has a larger leakage resistance and the surface impedance becomes closer to being modeled by an ideal capacitor. The surface capacitance gets smaller due to the increase in thickness. Furthermore, thicker monolayers block the access of the non-specific molecules from interfering with the measurements, which denotes that the thicker monolayer has a better “blocking capability”.

Another important consideration is the stability of monolayers as a function of time. We measured the drift in the surface impedance, specifically the changes in phase angle, to determine monolayer stability. An unstable monolayer shows a significant drop in the phase angle, which corresponds to lower surface coverage. We observed that the stability of monolayers of length greater than 10 carbon atoms (11-MUA in our case) is greater than a day but for monolayers less than 7 carbon atoms in length (3-MPA and thioctic acid in our case), the impedance starts to change even after a couple of hours. This indicates that longer monolayers are much more stable than shorter ones.

Even though longer monolayers are more stable and have better blocking capabilities, they typically have less transduction gain to molecular attachment. The binding sites are located farther away from the surface for a longer monolayer and hence the change in the interface impedance is lesser with binding. Furthermore, the longer monolayer presents a smaller series capacitance, which reduces the change in the overall capacitance with binding.

**Figure 10.**
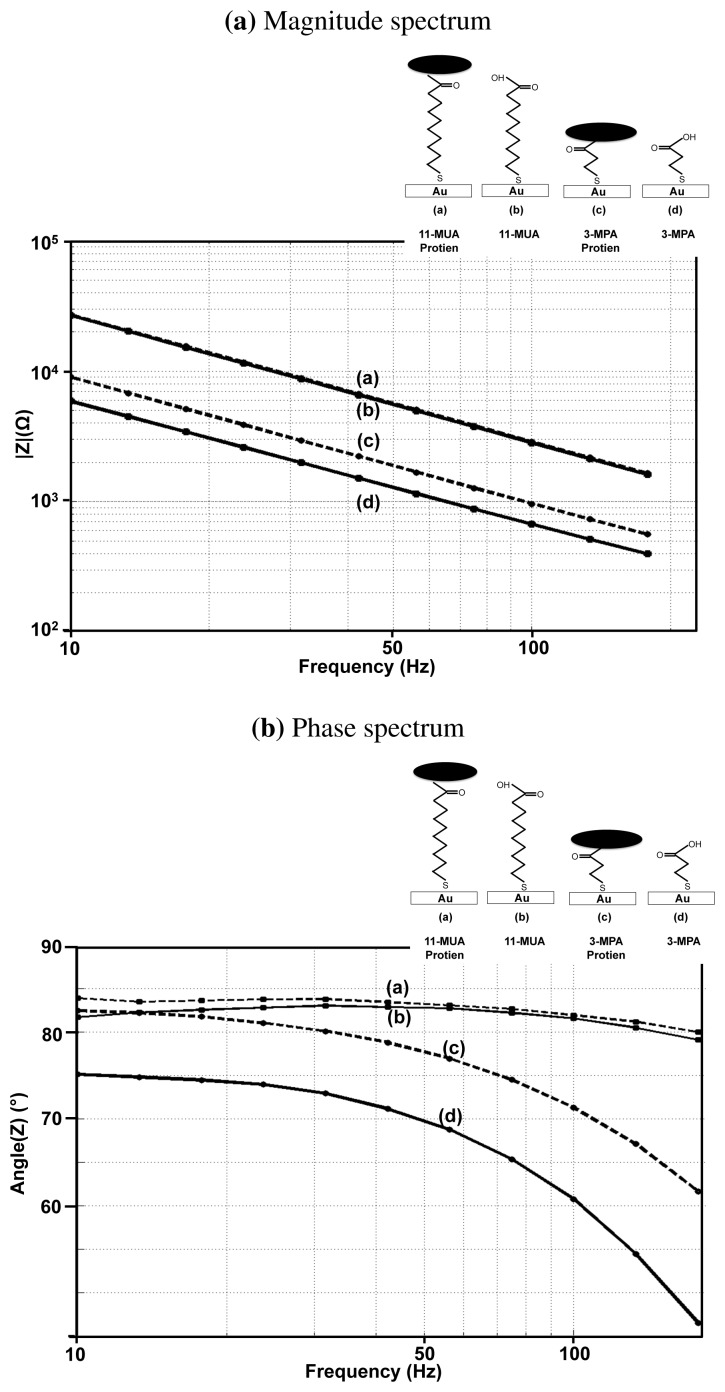
Experimental determination of magnitude and phase spectrum of (a) 11-MUA with protein attached (b) 11-MUA only (c) 3-MPA with protein attached (d) 3-MPA only.

In order to verify our theory, we performed experiments, in which the impedance is measured before and after the attachment of a protein molecule on the 11-MUA and 3-MPA monolayer. The protein molecule used in the experiments is FoS, which has a molecular weight of 78 kDa. The detection of FoS and anti-Fos binding is of great importance to neurologists, as it serves as a marker for neuronal activity [34]. The results obtained are shown in Figure 10(a,b).

As evident from the figure, the change in the surface impedance can be barely seen in the case of the 11-MUA monolayer, whereas the capacitance changes by 20% in the case of 3-MPA monolayer. A similar trend can be seen in the phase response where protein attachment causes only one degree change in the phase angle for the 11-MUA monolayer, whereas for 3-MPA, the phase angle changes by 4**°**. These experiments show that for a reasonable trade-off between the sensitivity on one side and the stability and blocking capability on the other, it is essential to choose linkers with intermediate length (7–8 carbon atoms).

### 4.3. Antibody Assays

To study the sensitivity of EIS based systems and to demonstrate its label-free capability and real-time nature, we built an antigen-antibody assay, which uses EIS for detection. Thioctic acid is chosen as the linker as it has the intermediate length of 7–8 carbon atoms. Thioctic acid has been previously used in [14] for building a protein-antibody assay and reasonable sensitivity has been demonstrated. We again selected FoS as our protein of interest. We studied the impedance changes that occurred firstly with the binding of anti-FoS IgG molecule to a FoS immobilized surface, followed by the binding of anti-IgG to an IgG immobilized surface. The detailed experimental procedure is provided in the supplementary material.

**Figure 11.**
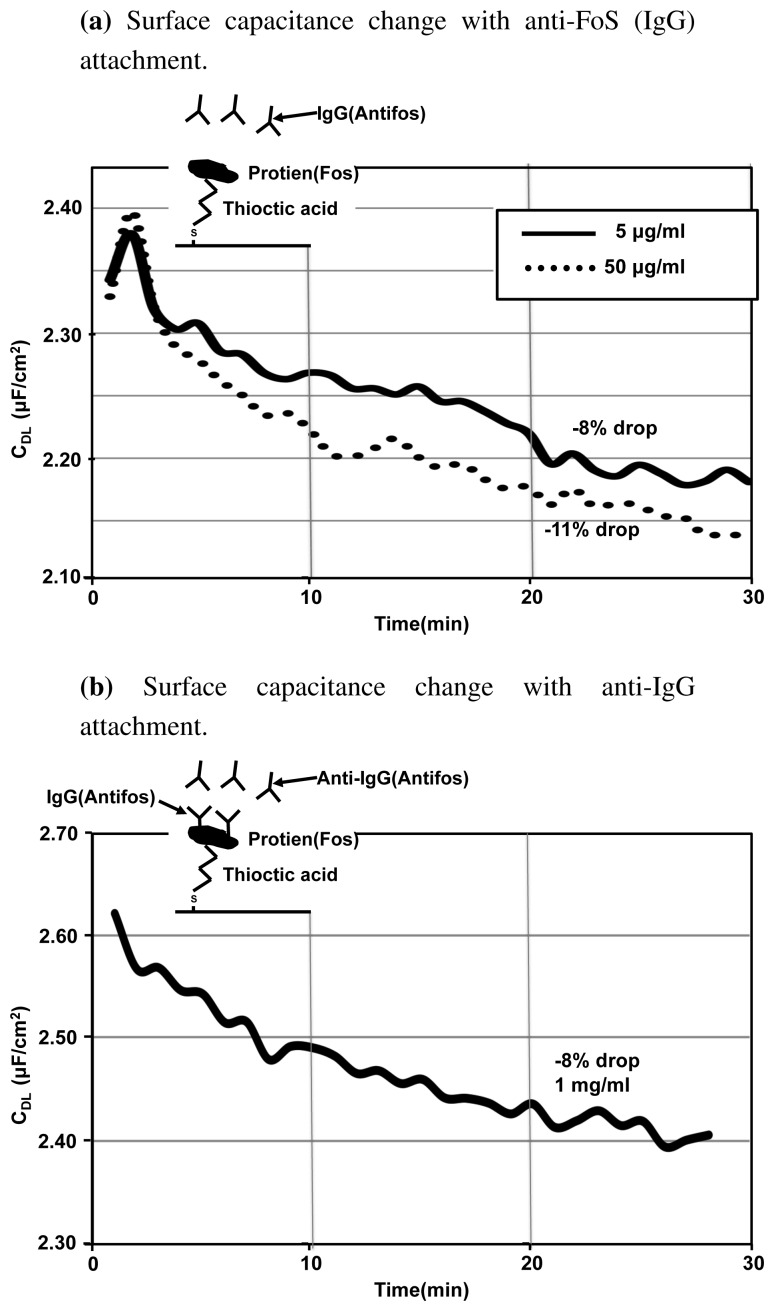
Real-time EIS study is done with addition of 5 $\frac{\mu g}{mL}$ and 50 $\frac{\mu g}{mL}$ anti-FoS (IgG) solution onto a slide that has FoS immobilized on it (**a**) and later with the addition of 1 $\frac{mg}{mL}$ anti-IgG solution (**b**).

Figure 11(a) shows the real-time experimental results that we obtained when antibody solution (anti-FoS IgG) is added to the 1 × PBS buffer. The slides immersed have FoS immobilized on a thioctic acid monolayer. We can see that the surface capacitance drops with the attachment of the antibody. There is a 11% change in the capacitance value with higher concentration $\left(50\;\frac{\mu g}{mL}\right)$ and 8% change with 10× dilution. As expected, the capacitance change is larger and capacitance value drops at a faster rate when the concentration of the antibody solution used is higher. The percentage change in capacitance is large, indicating that EIS has a reasonably good sensitivity.

Figure 11(b) shows the changes in the surface capacitance when anti-IgG is introduced on anti-FoS IgG immobilized surface. The capacitance changes by 8% in 30 min. Thus we can observe that the system is sensitive to an additional level of antibody attachment. EIS is a sensitive method for detecting biomolecular interactions happening close to the surface and is capable of detecting multiple levels of antibody attachment. EIS can provide a cheap and quick method for studying the kinetics of molecular attachment close to the electrode surface. Also another application of EIS is in studying the quality of monolayers formed. Any holes in the monolayer can translate to large changes in the surface impedance and hence we can perform some form of quality control on the immobilized monolayers using EIS.

## 5. On-Chip Experimental Measurements

### 5.1. Packaging and Setup

**Figure 12.**
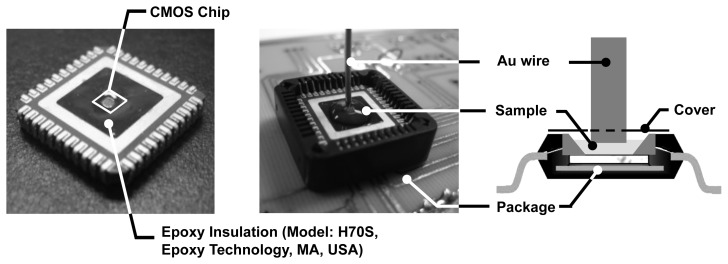
Packaging and electrochemical experimental setup.

Having developed optimized detection protocols, using macroscopic gold slides, we will now discuss the electrochemical and biomolecular experiments that we performed using our CMOS EIS chip. For performing EIS experiments, we need to bring the sensing electrode array in contact with the solution (electrolyte). To do this without interfering with the electronic data acquisition, we isolate the conductive solution from the bond-wires, I/O pads, and the IC package by using an electrically insulating epoxy (Epotek H70S), as shown in Figure 12. Subsequently, the 1mm diameter Au wire electrode, which serves as the reference, is immersed into the solution carefully on top of the sensing surface.

The experimental details that we obtained from our EIS chip are presented in [10] and the protocol is explained in the supplementary section. In this section, we will discuss some of the results that we obtained using the chip.

### 5.2. Real Time Experiment

**Figure 13.**
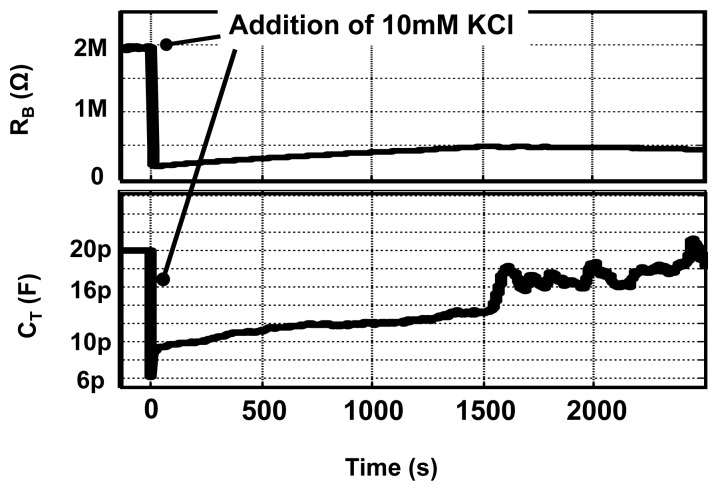
Real time control experiments.

Real-time detection is a very useful feature, which enables us to determine the kinetics of molecular interactions. We performed control experiments, using our EIS IC platform, in which the concentration of KCl solution is changed during an experiment as shown in Figure 13. Initially 120 *μL* of 1 mM KCl is present into which 6 *μL* of 10 mM KCl is added. The measurements results shown here are done at *ω* = 100 kHz and data points are collected with a rate of 1k sample/s. Shown in the figure, is the variation in the capacitance and resistance value with time on the addition of the 10 mM KCl solution. From the figure, it is possible to observe the kinetics of mixing process and calculate the time constant for settling. The real-time detection capability provided by our CMOS EIS sensor is significant, since only a few commercially available platforms offer this feature.

### 5.3. Impedance Spectra Measurements

**Figure 14.**
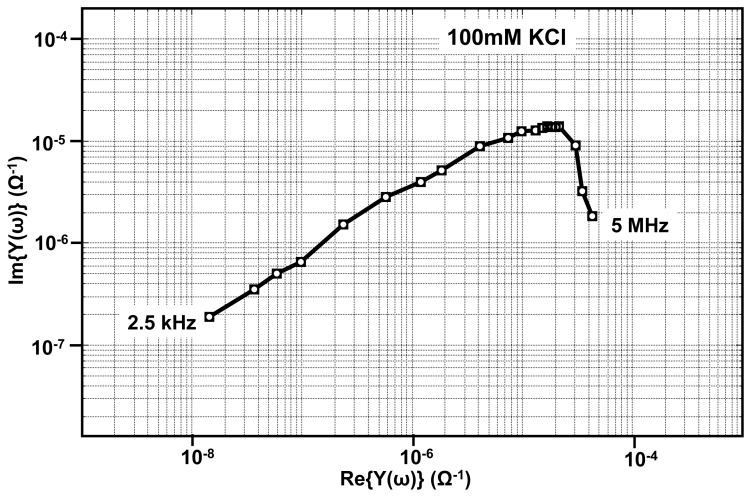
Admittance spectra of 100 mM KCl.

Apart from the real-time measurements, our EIS platform can be used for measuring impedance under equilibrium conditions. Shown in Figure 14, is one such admittance spectrum obtained using 100 mM KCl solution. The measurements are made with zero DC potential difference between the reference electrode and the sensing electrode and with a sinusoidal wave of small amplitude (10 mV) applied at different frequencies ranging from as low as 100 Hz to 50 MHz. In order to interpret the data, the admittance spectrum is fitted onto the equivalent circuit parameters as shown in the Figure 2. The fitting is done using LEVMW tool, which utilize complex non-linear least square-fitting algorithm [37].

**Figure 15.**
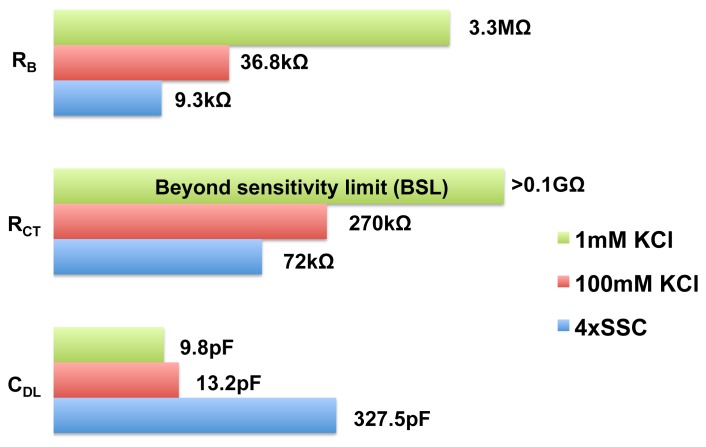
Equivalent circuit parameters for three different biological buffers. The experiments were done using on-chip electrodes.

Using our EIS IC, we performed impedance measurements with various biological buffers and the equivalent circuit parameters, which we obtained by fitting the experimental data, is shown in Figure 15. The buffers considered are 1 mM KCl, 100 mM KCl and 4× SSC. These buffers have a wide range of conductivities, 141 $\frac{\mu S}{cm}$, 14.1 $\frac{mS}{cm}$, 50 $\frac{mS}{cm}$. The magnitude *vs.* frequency and phase *vs.* frequency plots are provided in the supplementary material. As shown in the figure, the bulk resistance of the various buffers varied over a wide range. The surface capacitance is also a function of concentration, as seen in the figure. Since our EIS IC has a wide dynamic range (100 dB), it is capable of accommodating a wide variety of buffers. The wide dynamic range makes our EIS IC a suitable candidate for open-platform biosensor applications. Since our chip is not capable of resolving admittance smaller than 10 nS, any circuit parameter value larger than >0.1GΩ cannot be measured accurately and such parameters are marked as beyond sensitivity limit (BSL).

**Figure 16.**
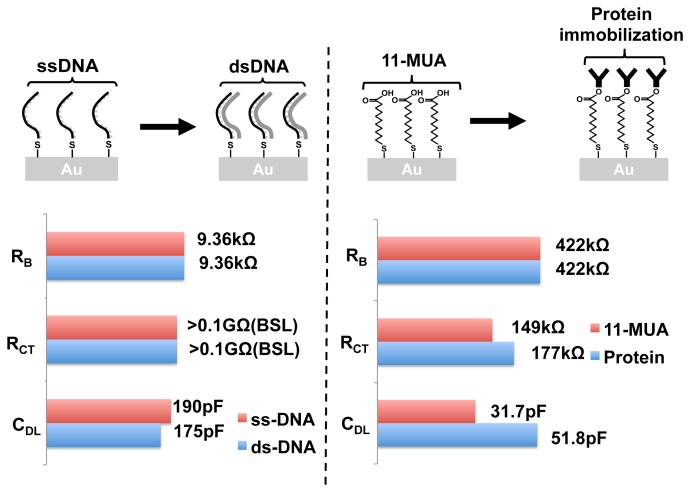
Equivalent circuit parameters for DNA hybridization experiment (**left**) and protein attachment detection (**right**). The experiments were done using on-chip electrodes. BSL refers to the beyond sensitivity limit.

DNA hybridization detection is key to all nucleic acid-based biosensors such as gene expression DNA microarrays. Using EIS, we can perform label-free and real-time DNA hybridization detection. A total of 10 frequency points were collected between 5 kHz and 300 kHz. The equivalent circuit parameters for immobilized ssDNA and hybridized dsDNA is shown in Figure 16. The bulk resistance *R_B_* stays the same, whereas the surface impedance changes. *R_CT_* values are larger than 0.1*G*Ω, and hence cannot be accurately measured by our chip. *C_DL_* drops by 8% with hybridization. This is an expected result as shown in other papers [39]. By linear extrapolation of this result, and based on our admittance sensitivity value, the detection limit of our system can be calculated to be around 10^5^ molecules, which is in par with other label-free methods reported [5].

Label-free detection of protein binding is of greater significance than DNA hybridization detection, as the binding capability of proteins (or antibodies) can change significantly with the attachment of labels. In our work, we performed label-free detection of protein attachment onto an 11-MUA monolayer. Ten frequency points were obtained in the frequency range of 500 Hz to 200 kHz. The equivalent circuit parameters of the monolayer and the change in impedance with the protein attachment are shown in Figure 16. We observed changes in *R_CT_* as well as *C_DL_*. As expected, no change was observed in the bulk resistance. Further studies need to be performed to better understand the changes in the admittance spectra. Nevertheless, EIS provides a method for studying protein binding without any additional molecular modifications. The magnitude *vs.* frequency and phase *vs.* frequency plots of the DNA hybridization and the protein attachment experiments are provided as a part of the supplementary material.

## 6. Conclusion

In this paper, we have described the different aspects of an EIS IC based system, with our EIS IC design as a reference. We have particularly focused on the interface design. We have justified the use of gold as the sensing electrode surface. We have discussed the important considerations in the interface design such as the attachment method and linker length and optimized the protocol for building a sensitive immunoassay using EIS. By making use of an example antigen-antibody biosensor system, we have demonstrated that EIS is a sensitive method, capable of detecting multiple levels of antigen-antibody binding.

We have presented a fully integrated EIS biosensor array, where the electrode and the detection electronics are integrated onto the same chip platform. The chip has a very wide dynamic range, capable of detecting impedance over five orders of magnitude and has a wide frequency range of operation. We have shown that it is possible to perform label-free and real-time studies of biomolecular interactions such as DNA and protein interactions using our EIS chip. We have shown that by taking advantage of IC fabrication processes, we can build a compact, high-performance biosensor array, which can push the detection of bio-molecular agents, from laboratories, which make use of bulky instruments onto miniaturized platforms that are usable in PoC diagnostic and other similar applications.

## Supplementary Material



## Figures and Tables

**Table 1.** Chip's key metrics.

Technology	0.35 *μm* CMOS, 4 metal layers, 3.3 V supply
Die size	2 *mm* × 2 *mm*
Array size	10 × 10
Pixel size	100 *μm* × 100 *μm*
Electrode	40 *μm* × 40 *μm*, Al/1% Si
Frequency range	10 Hz–50 MHz
Power consumption	84.8 mW (100 kHz)
Dynamic Range (BW = 10 Hz)	97 dB
