# Supplementary material for: Interface Design for CMOS-Integrated Electrochemical Impedance Spectroscopy (EIS) Biosensors
Source: Sensors (Basel). 2012 Oct 29;12(11):14467–88. doi: 10.3390/s121114467 (PMC3522923; doi:10.3390/s121114467)
Supplement: Supplementary file 1 [file sensors-12-14467-s001.pdf]

OPEN ACCESS

sensors

ISSN 1424-8220

www.mdpi.com/journal/sensors

Supplementary Information

Interface Design for CMOS-Integrated Electrochemical Impedance Spectroscopy (EIS) Biosensors. *Sensors* 2012, *12*, 14467-14488

Arun Manickam [1,2,*], Christopher Andrew Johnson [2], Sam Kavusi [2] and Arjang Hassibi [1]

[1] Department of Electrical and Computer Engineering, University of Texas at Austin, Austin, TX 78712, USA; E-Mail: arjang@mail.utexas.edu

[2] Robert Bosch Research and Technology Center, Palo Alto, CA 94034, USA; E-Mails: christopher.johnson2@us.bosch.com (C.A.J.); sam.kavusi@us.bosch.com (S.K.)

* Author to whom correspondence should be addressed; E-Mail: mmarun@utexas.edu; Tel.: +1-512-674-5023.

1. Antigen-Antibody Experimental Protocol

The protocol for the antigen-antigen experiments is summarized in Figure 18.

Figure S1. Protocol for protein/antibody experiment.

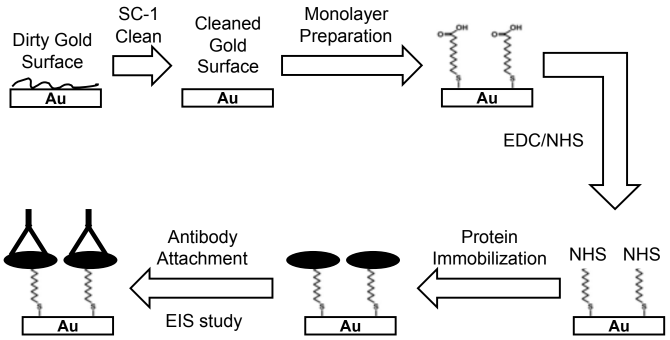

1.1. Surface Cleaning

1. The fresh gold slides are washed in acetone for 1 minute.
2. The slides are then placed in ethanol for 1 minute.
3. After drying under nitrogen gas, the slides are placed in an air plasma chamber (700 atm) for 90 seconds.
4. For SC-1 clean, a large glass beaker is filled with 1,000 mL of water. Inside the beaker, a smaller Teflon beaker containing 100 mL of DI water is placed.

5. The large glass beaker is placed on a hot plate and heated to 70 °C.
6. Once the temperature reaches 70 °C, 20 mL of ammonium hydroxide and 20 mL of hydrogen peroxide is added to contents of the Teflon beaker. The temperature is reduced to 65 °C.
7. The gold slides are placed in the Teflon beaker with the SC-1 mixture. One can observe vigorous bubbling on the gold surface. Warning: SC-1 mixture can cause severe irritation and damage to the skin. Hence it is essential to wear proper protection before using the mixture.
8. Leave the slides in the mixture for 15 minutes.
9. After the SC-1 clean, place the slides in a well-cleaned beaker containing DI water. Place it on the shaker for 10 minutes.
10. Transfer the slides to another beaker containing DI water. Place it on shaker for 10 minutes.
11. It is possible that a gold oxide layer might be formed on the gold surface. To reduce the oxide layer, place the slides in ultrapure ethanol and sonicate for 1 hour.

1.2. Monolayer Preparation

12. Prepare fresh solutions of 1 mM of alkanethiol in ultrapure ethanol.
13. Place the cleaned slides in a beaker containing the 1 mM solution. Back fill with nitrogen gas and tightly seal the beaker to make it airtight.
14. Place the beaker in a dark room for 24 hours.

1.3. EDC/NHS Protocol

15. Prepare a fresh solution of 75 mM EDC in ethanol and 25 mM NHS in 1× PBS.
16. Remove the slides from the monolayer preparation solution. Wash twice in ultrapure ethanol and dry it in nitrogen gas.
17. Place the slides in EDC/NHS solution for 4 hours. Backfill the beaker with nitrogen gas and place an airtight seal over the beaker.

1.4. Protein Immobilization

18. Prepare protein/antibody solutions in 1× PBS buffer.
19. Take the slides from the EDC/NHS solution beaker. Wash it twice in 1× PBS.
20. Wash it once in DI water and dry it under nitrogen gas
21. Pipette 7–10 µL of the protein/antibody solution onto the EDC/NHS treated gold slide. Use cover slips for the liquid to cover the required portions of the slide.
22. Place the slide in a humid chamber for 30 minutes.
23. At the end of 30 minutes, remove the cover slips and wash the slides in 1× PBS twice.
24. Place the protein immobilized slides in 100 mM sodium bicarbonate in 1× PBS (pH 8.0) buffer for 30 minutes. This step deactivates any reactive ester groups on the surface.
25. Place the slides in 1× PBS solution. The slides are now ready for EIS experiments.

1.5. Antibody Experiments

26. The gold slides with thioctic acid monolayer are first subject to EDC/NHS protocol (steps 15–17) and protein immobilization with a 200 nM FoS in 1× PBS solution (steps 18–25).
27. Place the slide in 33 nM anti-FoS in 1× PBS solution.
28. Study the attachment of anti-FoS onto a FoS immobilized surface in real-time using EIS.

    The EIS is performed using a bench-top Solartron impedance analyzer. A three-electrode setup is used, in which Ag/AgCl electrode serves as the reference electrode and a platinum wire serves as the counter electrode. A three-electrode setup is commonly used, as the potential of the bulksolution can be set accurately using the reference electrode, irrespective of the magnitude of the current flow through the counter electrode.

    Make measurements for 30 minutes and measure the impedance at 1, 5, 10, 50, 100, and 500 Hz.
29. After an hour, place the slides in 1 mg/mL of anti-IgG, which can attach to the immobilized anti-FoS IgG. Repeat real-time EIS measurements.

2. On-Chip DNA Hybridization Experiments

1. The fresh packaged chips are washed in acetone for 1 minute.
2. The packaged chips are then placed in ethanol for 1 minute.
3. After drying under nitrogen gas, the packaged chips are placed in an air plasma chamber (700 atm) for 90 seconds.
4. The chips are placed in 40 μL of 10 ng/mL of thiolated ss-DNA probe immobilization overnight. The buffer used 4× SSC with 5% glycerol.
5. The impedance is measured using on-chip detection circuitry in 4× SSC buffer.
6. 40 μL of 1 ng/mL in 4× SSC of complementary DNA strands is added. After 30 minutes, impedance is once again measured using on-chip detection circuitry.

3. On-Chip Protein Binding Experiments

1. The fresh packaged chips are washed in acetone for 1 minute.
2. The packaged chips are then placed in ethanol for 1 minute.
3. After drying under nitrogen gas, the packaged chips are placed in an air plasma chamber (700 atm) for 90 seconds.
4. Cover the chip with 40 μL of a glycerol/ethanol (1:1 v/v) solution containing 150 mM 11-MUA for at least 12 hours. Put a cover slip on top to prevent evaporation
5. Wash the chip in distiller water.
6. The impedance is measured using on-chip detection circuitry in 1× PBS buffer.
7. Cover the chip with a solution of 10% EDAC in water/ethanol (10:1 v/v) for 2 hours to activate the terminal COOH group of 11-MUA.
8. Wash the chip in distilled water. Cover the chip with 0.1mg/mL solution of protein-G containing 10% glycerol for 2 hours.
9. Wash the chip in distilled water. The impedance is measured using on-chip detection circuitry in 1× PBS buffer.

4. On-Chip Admittance Measurements

Following are the experimental results obtained using our CMOS EIS sensor. The on-chip 40 µm × 40 µm electrodes were used as the sensing interface and the impedance was measured using the on-chip pixel circuitry.

(a) Experiments with KCl buffers with three different concentrations (1 mM, 10 mM and 100 mM)

Figure S2. (**a**) Magnitude *vs.* Frequency; (**b**) Phase *vs.* Frequency.

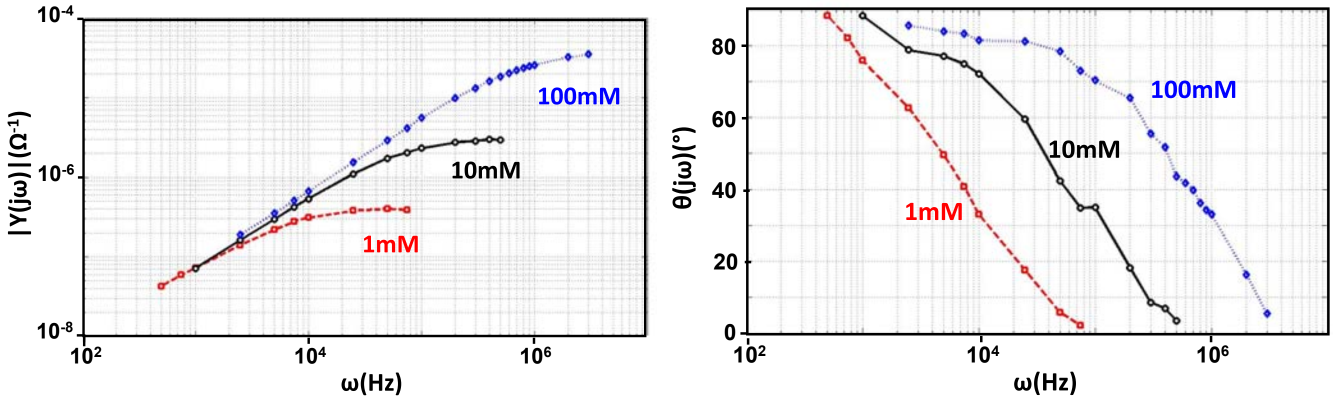

(**a**) (**b**)

(b) DNA Hybridization experiments

Figure S3. (**a**) Magnitude *vs.* Frequency; (**b**) Phase *vs.* Frequency. Solid line: ss-DNA Dotted line: ds-DNA.

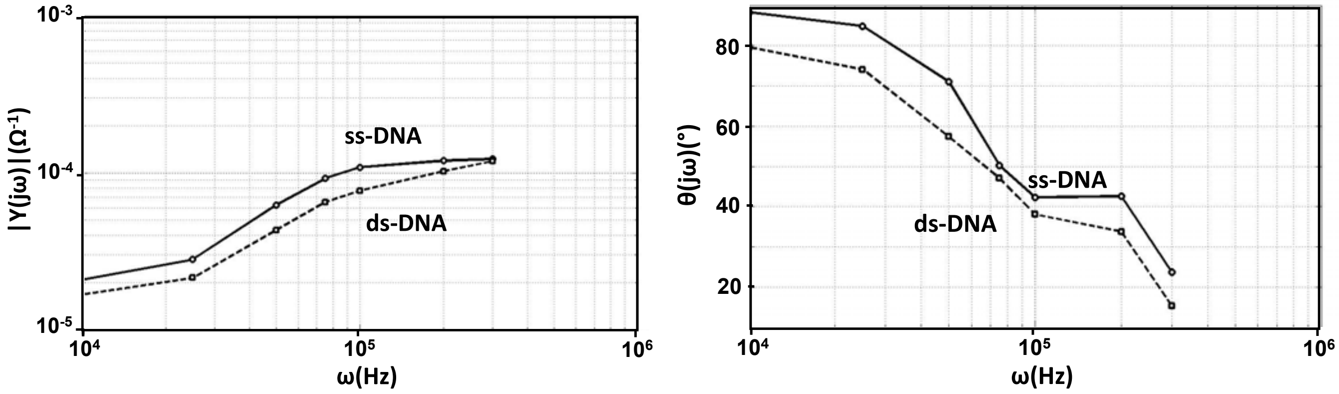

(**a**) (**b**)

(c) Protein attachment experiments

Figure S4. (**a**) Magnitude *vs.* Frequency; (**b**) Phase *vs.* Frequency. Solid line: 11-MUA only Dotted line: 11-MUA + Protein-G.

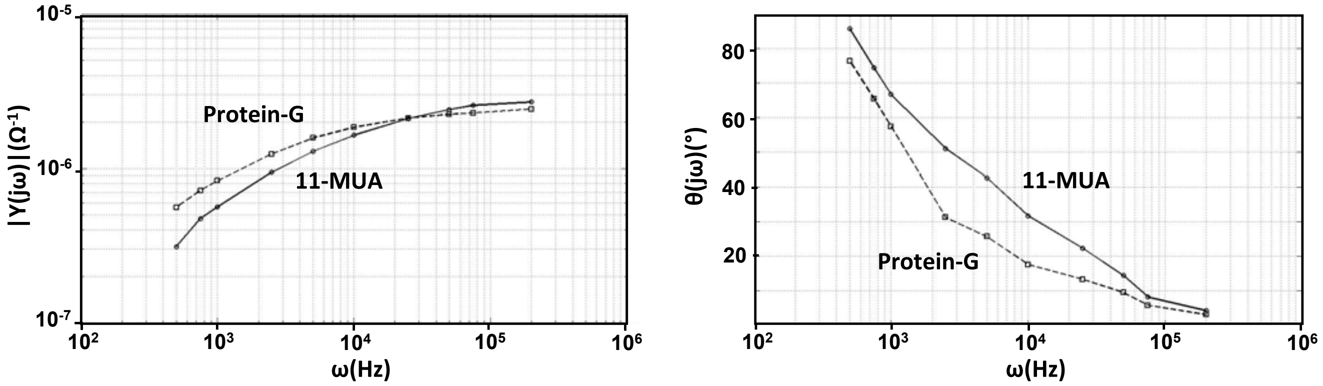

(**a**) (**b**)

© 2012 by the authors; licensee MDPI, Basel, Switzerland. This article is an open access article distributed under the terms and conditions of the Creative Commons Attribution license (http://creativecommons.org/licenses/by/3.0/).
